# Impact of Hydroxyapatite on Gelatin/Oxidized Alginate 3D-Printed Cryogel Scaffolds

**DOI:** 10.3390/gels10060406

**Published:** 2024-06-18

**Authors:** Ainur Zhanbassynova, Fariza Mukasheva, Madi Abilev, Dmitriy Berillo, Alexander Trifonov, Dana Akilbekova

**Affiliations:** 1 Department of Chemical and Materials Engineering, School of Engineering and Digital Sciences, Nazarbayev University, Astana 010000, Kazakhstan; ainur.zhanbassynova@nu.edu.kz (A.Z.);; 2 Department of Chemistry and Biochemical Engineering, Satbayev University, Almaty 050013, Kazakhstan

**Keywords:** 3D printing, cryogelation, hydroxyapatite, stem cells, bone tissue engineering

## Abstract

Fabrication of scaffolds via 3D printing is a promising approach for tissue engineering. In this study, we combined 3D printing with cryogenic crosslinking to create biocompatible gelatin/oxidized alginate (Gel/OxAlg) scaffolds with large pore sizes, beneficial for bone tissue regeneration. To enhance the osteogenic effects and mechanical properties of these scaffolds, we evaluated the impact of hydroxyapatite (HAp) on the rheological characteristics of the 2.86% (1:1) Gel/OxAlg ink. We investigated the morphological and mechanical properties of scaffolds with low, 5%, and high 10% HAp content, as well as the resulting bio- and osteogenic effects. Scanning electron microscopy revealed a reduction in pore sizes from 160 to 180 µm (HAp-free) and from 120 to 140 µm for both HAp-containing scaffolds. Increased stability and higher Young’s moduli were measured for 5% and 10% HAp (18 and 21 kPa, respectively) compared to 11 kPa for HAp-free constructs. Biological assessments with mesenchymal stem cells indicated excellent cytocompatibility and osteogenic differentiation in all scaffolds, with high degree of mineralization in HAp-containing constructs. Scaffolds with 5% HAp exhibited improved mechanical characteristics and shape fidelity, demonstrated positive osteogenic impact, and enhanced bone tissue formation. Increasing the HAp content to 10% did not show any advantages in osteogenesis, offering a minor increase in mechanical strength at the cost of significantly compromised shape fidelity.

## 1. Introduction

Bone tissue engineering (BTE) represents a progressive therapeutic approach for reconstructing bone tissue in significant bone defects, bone loss, fractures, or conditions such as arthritis, osteoporosis, and osteosarcoma [1]. Bone tissue is a complex and dynamic structure that plays a crucial role in supporting and protecting the body. Bone consists of an outer dense cortical layer and an inner porous trabecular layer; the cortical bone provides strength and rigidity, while the trabecular bone, with its spongy morphology, offers lightweight support and aids in shock absorption. Hydrogel scaffolds are designed to mimic this structure by having a solid framework that resembles the strength of cortical bone and a porous network that replicates the nutrient-conductive and cell-supportive properties of trabecular bone. This similarity ensures that hydrogel scaffolds can support cellular activities and mechanical loads, facilitating effective bone tissue regeneration. The fundamental objective of BTE is to foster tissue regeneration and new bone growth using degradable polymeric scaffolds as replacement matrices to fill bone defects and facilitate cell proliferation and regeneration. Ideally, scaffolds should possess sufficient rigidity to provide structural support during the regeneration process and allow applications in load-bearing bones. Scaffolds are predominantly based on synthetic or natural polymers or their composites, and function as artificial extracellular matrices for osteoblasts or stem cells capable of differentiating into osteoblasts [2]. The core criteria that hydrogel scaffolds should satisfy are low cytotoxicity and biodegradability to allow sufficient time for cell attachment, proliferation, growth, and extracellular matrix (ECM) deposition as new bone tissue is formed [3]. Ideally, the rates of scaffold degradation and tissue formation should match. Matrix morphology, particularly its porosity characteristics, is a key feature in hydrogel scaffolds which can define the rate and efficiency of multiple biological processes. Hydrogel matrices with pore sizes greater than 100 μm support better bone and capillary growth by allowing unobstructed cell infiltration and growth [4]. Larger pore sizes enhance the supply of oxygen and nutrients, resulting in increased alkaline phosphatase (ALP) activity and calcification during the osteo-differentiation of stem cells [5].

Along with advantages suggested by synthetic polymers, such as ease of manufacturing, tunable properties, and superior mechanical strength, in recent years, natural polymers have garnered attention as promising candidates for scaffold materials, owing to their high biocompatibility, adaptability, and capacity to emulate the native tissue’s ECM, which is predominantly composed of collagen [4,6]. Gelatin (Gel) is a collagen derivative that is among the most popular natural polymers used in tissue engineering [7]. Nonetheless, the mechanical characteristics and temporal integrity of gelatin in isolation often fall short of specific applications, necessitating modifications to improve its robustness. Oxidized alginate (OxAlg) has emerged as a promising additive for gelatin-based scaffolds, acting as both a building and crosslinking element owing to its multiple reactive functions, including carboxylic acids and aldehydes [8]. Whereas aldehydes engage in condensation reactions with the free amines in lysine or hydroxylysine residues of gelatin [9,10], carboxyl groups can interact with divalent ions, creating an additional crosslinking effect [11]. Both covalent and electrostatic interactions contribute to the establishment of a chemically robust polymeric network with excellent biocompatibility.

A variety of 3D-printing techniques have been used for the fabrication of scaffolds for tissue engineering, including powder bed fusion, material or binder jetting, vat photopolymerization, directed energy deposition, lamination, and extrusion [12]. The viscoelastic properties and stabilizing interactions of the Gel/OxAlg mixture allowed this composition to be adapted for extrusion-based 3D-printing and bioprinting fabrication processes, demonstrating a fast and cost-effective fabrication approach using computer-aided design (CAD) [13,14,15,16,17]. Modern 3D printers provide high-precision control over multiple parameters, granting extensive flexibility and on-demand fine-tuning during fabrication. Although geometry is defined by a CAD file, the shape fidelity and integrity of the scaffold can be controlled by optimizing the extrusion pressure, temperature, and deposition speed [18,19]. Physical characteristics such as porosity, swelling capacity, mechanical properties, and degradation rate can be regulated by controlling the polymer concentration and fabrication conditions [20,21,22,23]. For example, dilution of ink or lowering the content of the crosslinker lead to the formation of larger pores but reduce the mechanical properties and temporal stability of the resulting scaffold.

Large pores have been shown to play a crucial role in bone tissue regeneration by providing conditions for effective mass transport and sufficient room for cell proliferation and extracellular matrix (ECM) deposition [23,24,25]. To achieve such pore dimensions, we employ 3D printing along with cryogenic synthesis to create biocompatible and mechanically robust porous hydrogel scaffolds. Optimization of the printing parameters allows for the fabrication of mechanically stable scaffolds with a range of pore diameters exceeding 200 μm. Such pore dimensions are achieved owing to the separation of polymer–water phases at sub-zero temperatures, creating ice-crystal-rich regions that provide a template for interconnected porous morphology as slow crosslinking occurs, revealing large pores after thawing. This approach is advantageous as it not only eliminates the need for pore-forming agents [24], but also allows the fabrication of interconnected porosity on sub-mm scale on the 3D-printed scaffold without compromising on robustness and mechanical stability.

Despite the immense potential of the combined fabrication approach and materials, Gel/OxAlg hydrogel scaffolds exhibit relatively low mechanical strength. This issue is particularly pronounced in scaffolds with large pores, making them unsuitable for load-bearing applications. Consequently, their use is restricted to the fabrication of scaffolds for in vitro models only [25,26]. A viable strategy to augment the mechanical robustness of these composites is to incorporate an inorganic phase, such as glass beads or hydroxyapatite (HAp) nanoparticles [27]. It has been reported that adding HAp markedly enhances the mechanical robustness of the scaffold, promotes osteogenesis and mineralization, and enhances bone regeneration capabilities [28].

Recently, various research groups have explored the integration of inorganic nanoparticles into Gel/OxAlg hydrogels for reinforcement. Hasan et al., utilized alginate-di-aldehyde cross-linked gelatin/nano-hydroxyapatite scaffolds formed by lyophilization. The authors reported high viability of normal kidney epithelial Vero cells; however, no bone model with calcium deposition and extracellular matrix development was achieved [29]. Similarly, Emami et al., reported that gelatin/oxidized alginate hydrogels with bisphosphonate-modified hydroxyapatite particles demonstrated improved mechanical properties but showed low viability of L-929 cells (64.1%) and no ECM mineralization [30]. Monavari et al., demonstrated that 3D-printed alginate dialdehyde-gelatin (ADA-GEL) hydrogels incorporating phytotherapeutic icariin-loaded mesoporous SiO_2_-CaO nanoparticle-enhanced osteoblast proliferation, adhesion, and differentiation. However, lower cell viability (40–60%) and rapid degradation (40% within the first 24 h) did not permit proceeding to assessment of the later stages of tissue formation [31].

In our previous studies [25,26], we demonstrated the fabrication of Gel/OxAlg hydrogel scaffolds with highly porous interconnected matrix by coupling 3D printing and cryogenic synthesis. By systematically optimizing the ratio and concentration of polymers along with printing parameters, we demonstrated that 2.86% (1:1) Gel/OxAlg ink can fabricate mechanically robust scaffolds with pores ranging between 80 and 280 µm in diameter. We found that minor decreases of as low as 0.2% *w*/*v* of polymer content lead to notable defects in integrity and relatively rapid degradation of scaffolds. In this study, we focused on improving the mechanical characteristics and bioactivity properties of the 2.86% Gel/OxAlg scaffolds by doping the precursor ink with low and high concentrations of HAp. We examined the rheological properties of the inks and compared the mechanical, morphological, and biological properties of the scaffolds while prioritizing printability and large pore sizes.

## 2. Results and Discussion

### 2.1. Biopolymer Ink Formulations and Rheological Characterization of Gel/OxAlg/HAp Cryogel Scaffolds

FTIR spectroscopy was used to verify the successful oxidation of sodium alginate and to characterize the products. A comparison of the spectra revealed a new peak at 1730 cm^−1^ in the oxidized product, typical of the symmetrical vibration of the aldehyde group, confirming the successful modification of the polymer and purity of the OxAlg compound (Figure S1). In Gel/OxAlg inks, gelatin primarily acts as the structural backbone, providing the shear-thinning properties necessary for efficient printing. In contrast, OxAlg facilitated major chemical crosslinking and ionic-stabilizing interactions. To assess the impact of HAp on physical and biological characteristics of the 3D-printed scaffold, produced from the optimized 2.86% Gel/OxAlg ink, two HAp-containing compositions were formulated: 5% and 10% w/dry polymer mass as low and high content, respectively, and compared to the HAp-free constructs (Table 1).

There are two main reasons for selecting the 5% concentration as low-content and 10% as high-content HAp compositions. Aiming to achieve significant improvement in scaffold rigidity and gain osteogenesis, a lower value was suggested to introduce notable changes in the mechanical characteristics of the scaffolds with minimal impact in viscoelastic properties and printability of the ink. The upper value was imposed by the capacity of the 2.86% Gel/OxAlg ink to retain a stable and homogeneous structure. Experimentally, we found that 12.5% HAp/Gel/OxAlg ink loses its homogeneity within 10–15 min or immediately upon applying pressure during extrusion, which practically leads to a loss of printability. Also, higher HAp contents of 15% and 20% were tested but deemed unsuitable for 3D printing. Therefore, a 10% HAp/Gel/OxAlg, which exhibited endurable stability and decent printability, was selected as the high HAp-content ink.

**Figure 1:**
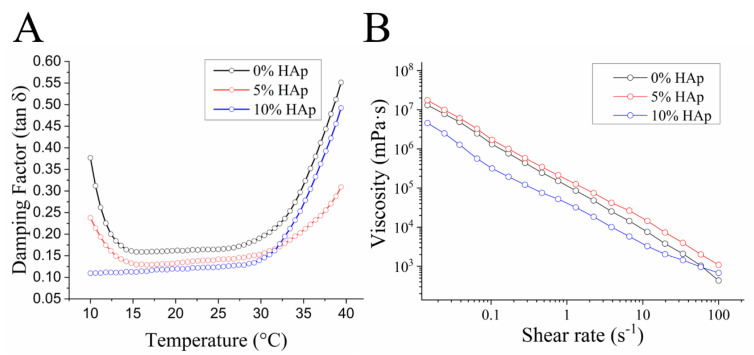
Rheological characterization of 2.86% Gel/OxAlg inks with 0%, 5%, and 10% HAp nanoparticles. (**A**) Damping factor (tan δ) vs. temperature; (**B**) flow curves of viscosity vs. shear rate (logarithmic scale) at 15 °C.

All three hydrogel compositions, 0%, 5%, and 10% HAp, underwent rheological characterization to verify the feasibility of extrusion-based 3D printing and to optimize the printing conditions. Figure 1A demonstrates a dynamic mechanical analysis of the inks, where the damping factor (tan δ) is calculated from the loss-to-storage moduli ratio, which indicates the balance between the viscous and elastic components of behavior as a function of temperature. The tan δ curves of all three compositions are typical for gelatin-based hydrogels and indicate predominantly elastic behavior in the 15–30 °C range, followed by a notable increase at higher temperatures. The relative values of tan δ indicate that energy dissipation decays with higher HAp content, demonstrating an enhanced gel-like behavior. The 5% HAp/Gel/OxAlg ink demonstrated a more gradual ascent that started at ca. 30 °C, thus signifying a tendency toward a higher transition temperature than the 0% and 10% HAp formulations. In contrast, 10% HAp/Gel/OxAlg ink tends to be more elastic at lower temperatures, extending the dominating effect of storage modulus range below 15 °C.

Figure 1B displays rheograms of the inks tested at 15 °C, representing the tan δ minimum. All ink compositions exhibited slopes around −45°, indicating shear-thinning behavior characterized by decreased viscosity as the shear rate increased. Notably, there was no pronounced transition from a solid-like to a fluid-like state at lower shear rates. The absence of such a transition is attributed to the viscoelastic effect, which may obscure the yield stress behavior. This effect is evident in the 10% HAp curve, where the slope changes to a less steep angle after ca. 0.1 s^−1^, indicating a complex behavior of the gels. The flow curves indicate that 5% HAp doping slightly increased the viscosity of the 2.86% Gel/OxAlg ink, mainly at higher shear rates, suggesting a more gel-like structure. The highest viscosity values of 5% HAp ink at low shear rates may indicate improved shape fidelity, whereas higher viscosity at higher shear rates suggests an increased extrusion pressure for smooth filament deposition of this composition. The increase in viscosity can be partially attributed to the adsorption of water by HAp particles, which leads to a higher polymer concentration and stabilizing ionic and hydrogen bond interactions between the HAp particles and biopolymeric chains. In contrast, doping of the ink with 10% HAp leads to a notable decrease in viscosity at low and intermediate shear rates, with a slight mitigation between 30 and 100 s^−1^. The reduced viscosity of the ink is attributed to a high concentration of well-dispersed nanoparticles, which reduces the overall resistance to flow, with possible gel structure reorganization at higher shear rates, facilitating a more efficient packing of HAp.

Although rheometry data supported the feasibility of using the tested inks for 3D printing, two interesting observations can be pointed out: (a) doping of HAp increases the viscosity of the ink at moderate concentrations and decreases at high, which indicates the existence of stabilizing and destabilizing interactions at different concentrations of dopant, and (b) HAp affects the viscoelastic properties of the ink and contributes to its elastic behavior, although the concentration of the HAp dopant seems to have a significant impact on different temperature domains: leading to an increase in elasticity below 15 °C for 10% HAp composition and above 30 °C for 5% HAp ink.

### 2.2. Fabrication and Mechanical Characterization of Gel/OxAlg/HAp Scaffolds

**Scheme 1:**
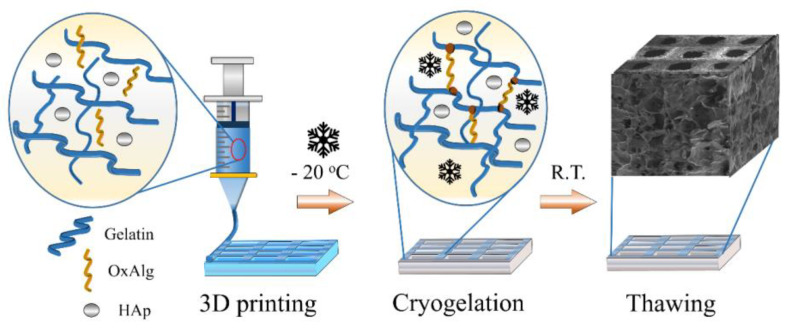
Fabrication of 3D-printed Gel/OxAlg-based hydrogel scaffolds (with or without HAp) via cryogelation. The process involves 3D printing of scaffolds using layer-by-layer ink deposition in a selected design, subjection of 3D-printed scaffold to −20 °C for 24 h for cryopolymerization, and thawing.

The fabrication process of the magnoporous [32] Gel/OxAlg-based scaffolds (interconnected pore diameters d > 100 µm) is depicted in Scheme 1. Following consecutive deposition of 10 layers of the respective ink in a checkered 10.1 × 10.1 × 2.25 mm pattern at optimized conditions, the produced scaffolds are subjected to subzero temperatures for cryogenic crosslinking for 24 h. After thawing, the obtained mechanically robust hydrogel scaffolds can be used or freeze-dried and stored at 4 °C for later use.

**Figure 2:**
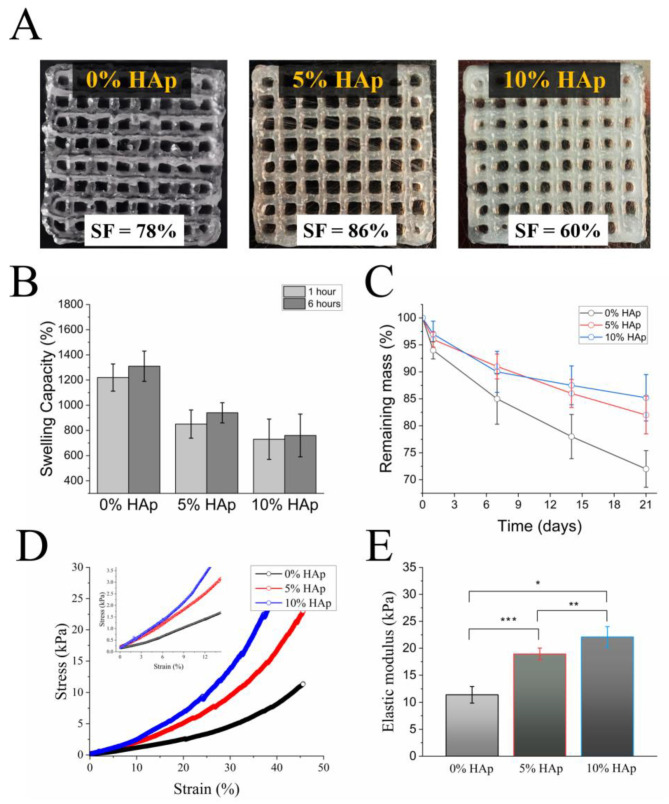
Characterization of the 3D-printed 2.86% Gel/OxAlg scaffolds produced with 0%, 5%, and 10% HAp. (**A**) Optical images of the scaffolds immediately after the printing with calculated SF (N = 4); (**B**) swelling capacity calculated for scaffolds after 1 and 6 h; (**C**) degradation rate of the scaffolds in PBS, at 37 °C; (**D**) compressive stress–strain behavior, with magnified region in the inset (for the elastic modulus calculation); and (**E**) Calculated compression modulus of the scaffolds (N = 3), considering range of 0–10% strain. Statistically significant difference is indicated through the symbols *, **, and *** according to the *p* < 0.05.

To assess the impact of HAp on 2.86% Gel/OxAlg ink printability and shape fidelity (SF) of the resulting scaffold, we used individually optimized printing settings for each composition to produce fibers of identical thickness. Comparative mass analysis of 75 scaffolds/ink compositions revealed deviations below 4.5% for 0% and 5% HAp, and below 6.6% for 10% HAp scaffolds. The lower mass precision of 2.86% Gel/OxAlg/10% HAp scaffolds is attributed to the relative changes in viscosity at low and high shears, which leads to imprecisions in the volume extruded at the start and the end of the printing process. The optimized printing parameters (Table 2) demonstrate that a higher HAp content in the ink increases the initial extrusion pressure required for printing, which correlates with the previously discussed rheological data. Figure 2A shows optical images of freshly printed 0%, 5%, and 10% HAp-containing scaffolds. We calculated the SF of the scaffolds by comparing the dimensions of the voids in the resulting scaffold with those specified by the CAD model. The obtained values indicate that 5% HAp doping improved the shape fidelity of the scaffold from 78 ± 5.2% to 86 ± 4.7% compared to the undoped composition. To ensure the optimal SF of the scaffolds, the printing bed temperature was set to 15 °C to achieve the minimal value of tan δ for each composition and to enhance the elastic behavior of hydrogel after deposition. The increased SF of the 5% HAp-containing scaffold correlates with the obtained rheological measurements, demonstrating higher viscosity at low shears. The same relation between low viscosity of the ink and reduced SF was observed for the 10% HAp scaffolds. Whereas the extrusion of 10% HAp ink requires higher pressure, its reduced viscosity at lower shears negatively affects the scaffold’s capacity for shape retention, which leads to reduced printing precision. A higher deposition speed was used to produce even filaments of 0.45 mm to compensate for lower viscosity. As a result, scaffolds produced from 10% HAp demonstrated significantly impaired precision and shape fidelity (SF = 60 ± 6.8%). The impact of HAp particles on viscosity and the resulting printing accuracy of hydrogels has been previously report [33,34] for Gel/Alg/HAp hydrogels, which is in line with our observations.

The physical characteristics of the scaffold, including its capacity to absorb bodily fluids, define vital parameters for tissue growth, such as cell proliferation, nutrient/waste transport, degradation, and ECM deposition rates [35]. The duration of bone tissue formation greatly depends on the cell type, scaffold characteristics, and conditions provided, and can last from a few weeks to several months to achieve full maturation [36,37]. The scaffold degradation rate should ideally synchronize with the rate of new tissue formation, with the initial two to three weeks being particularly critical as cells proliferate, activating biological mechanisms for ECM secretion and the start of mineralization. During this period, the mechanical stability of the scaffold is essential to support efficient cell seeding and proliferation [38,39]. Figure 2B depicts the swelling capacity (SC) profiles of 0%, 5%, and 10% HAp-containing Gel/OxAlg scaffolds immersed in PBS and recorded after 1 and 6 h. It was observed that all scaffold variants exhibited a substantial increase in swelling capacity: 1220%, 850%, and 730% after 1 h immersion for 0%, 5%, and 10% HAp-containing scaffolds, respectively. After 6 h, a 7% increase in swelling mass was recorded for 0% and 5% HAp scaffolds, whereas scaffolds with 10% HAp demonstrated negligible changes. Notably, 5% HAp scaffolds demonstrated the lowest deviation in swelling capacity, which correlated with our calculations of the lowest deviation in SF for these scaffolds. The same correlation was observed for the 0% and 10% HAp scaffolds. Despite the notable difference in SC between the HAp-containing and HAp-free constructs, this gap is mitigated by the fact that HAp contributes to the total mass of the scaffolds and, thus, reduces the relative mass changes upon absorption of an equal amount of liquid, despite the capability of HAp for additional absorption as compared to the HAp-free constructs.

The swollen scaffolds were immersed in buffer, and their masses were monitored for 3 weeks. Figure 2C summarizes the results of the degradation experiments. Scaffolds with 5% and 10% HAp demonstrated a notably detained degradation rate, retaining 82.0 and 85.2% of their initial dry mass, respectively, after three weeks. No statistically significant differences were observed between the two scaffold types throughout the test. HAp-free scaffold degraded faster and retained only 72% of its initial dry mass. Significant variability in degradation between the HAp-containing and HAp-free scaffolds was noted from day 1 and continued throughout the experiment. Steady yet detained degradation of 5% and 10% HAp scaffolds suggests their higher resistance to hydrolysis and mechanical robustness, which can be explained by morphological differences (e.g., porosity, interconnectivity) in hydrogel matrices caused by HAp particles.

To assess the impact of HAp on the robustness and mechanical qualities, the fabricated scaffolds were subjected to a stress–strain test, Figure 2D, and the elastic modulus for each type of scaffold was calculated, Figure 2E. The results indicate that the stiffness of scaffolds grows with a higher content of HAp, and calculated values of elastic modulus demonstrated an increase from 11 ± 0.15 to 18 ± 0.11 kPa for 5% HAp and a further increase to 21 ± 0.22 kPa at 10% HAp concentration. Although HAp nanoparticles significantly improved the mechanical characteristics and contributed to the overall stability of the scaffold, the obtained stiffness values are significantly lower than those of native cortical bone (compression strength of ~200 MPa). This means that HAp on its own can scarcely lead to further significant improvement in mechanical strength to achieve the MPa-GPa range and additional approaches to increase elasticity and robustness of the scaffolds should be considered.

Previous reports have indicated a similar impact of HAp on the mechanical properties of hydrogel scaffolds [40]. In one of the studies, chitosan-based cryogels with 20% HAp exhibited a significantly higher elastic modulus (2.012 ± 0.328 MPa) compared to cryogels without HAp (10.8 ± 0.47 kPa) [41]. In [42], HAp in alginate-based hydrogels significantly increased the compressive stress at small deformations, with 6% HAp being the optimal concentration. However, such notable improvements were achieved by using significantly higher concentrations of biopolymers, resulting in scaffolds with smaller pore diameters and better mechanical properties.

### 2.3. Morphological Characterization

**Figure 3:**
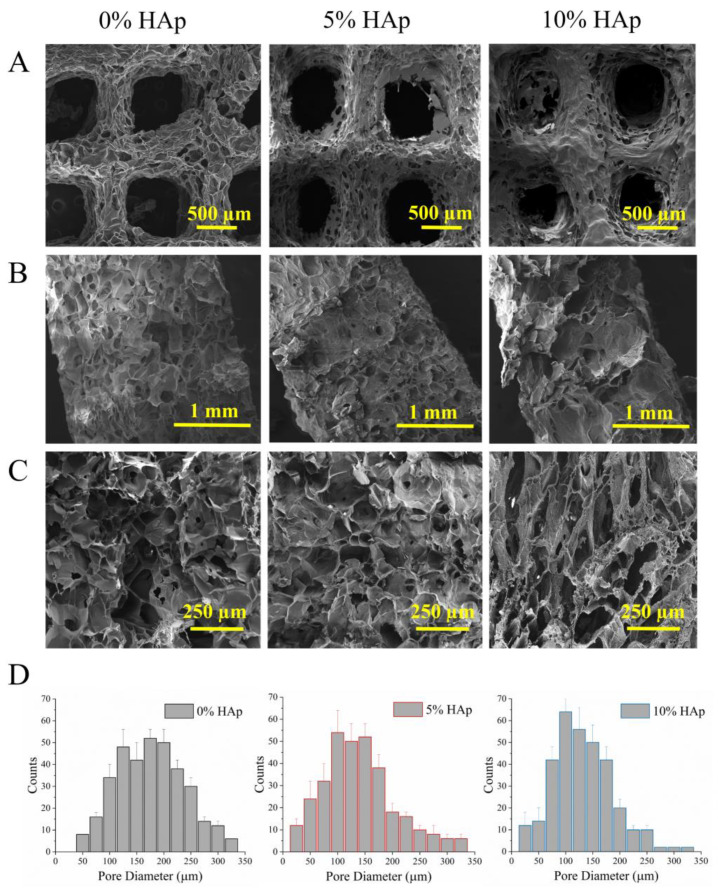
Morphological characterization of the 3D-printed 2.86% Gel/OxAlg scaffolds with 0%, 5%, and 10% HAp. SEM images of (**A**) top view; (**B**) cross-sectional view; (**C**) magnification of representative region; (**D**) pore size distribution histograms calculated for each type of scaffold.

Scanning electron microscopy (SEM) analysis was conducted to ascertain the impact of HAp concentration on the morphology of the hydrogels. The samples were cryogelated and lyophilized prior to SEM imaging. Figure 3A–C show the SEM images of the top, cross-sectional, and magnified views of the fabricated scaffolds. The SEM micrographs revealed that the structural integrity of all scaffolds was preserved throughout the fabrication and post-production stages. Each composition yielded a homogeneously distributed, highly interconnected porous structure. It was noted that 10% HAp samples revealed a smoother surface and reduced thickness of the constructs, which were attributed to a lower shape fidelity for this composition. An additional notable difference was the pore morphology of the high-HAp samples, which exhibited more elongated and stretched shapes. In pore size analysis, Figure 3D shows that the symmetrical distribution of pore sizes of 0% HAp scaffolds was shifted to a right-skewed Gaussian in the case of 5% HAp. Due to the elongated shape of the 10% HAp sample pores, average diagonal pore dimensions were used for pore size distribution plotting. Therefore, we cannot unambiguously claim the same right-skewed distribution for the 10% HAp scaffolds despite the same notable shift in pore sizes. An average 174 ± 61 µm pore diameter was measured for the 0% HAp, whereas pore sizes for the 5% and 10% HAp showed lower average dimensions: 151 ± 71 µm and 143 ± 67 µm. This interesting observation suggests that HAp doping contributes to the homogeneity of the ink and reduces the efficiency of the polymer–water phase separation during the cryogelation step, which leads to a reduction in the average pore diameter.

It should be noted that the decrease in average pore sizes, and thus, the higher surface area of the polymer backbone structure can be associated with the detained degradation rates of HAp-containing scaffolds.

### 2.4. In Vitro Characterization: Osteogenic Differentiation of Rat MSCs and ECM Mineralization

**Figure 4:**
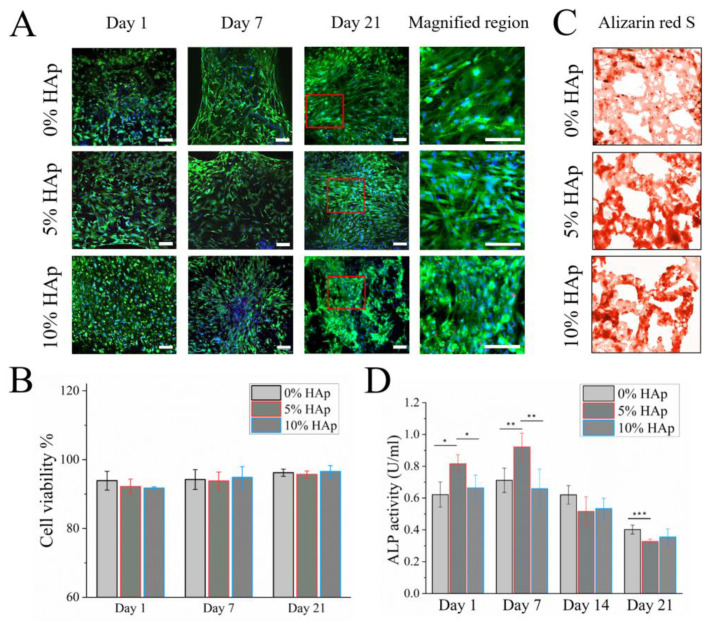
In vitro evaluation of 3D-printed 0%, 5%, and 10% HAp Gel/OxAlg scaffolds seeded with rat MSCs. (**A**) Representatives merged live/dead staining images of the scaffolds on days 1, 7, and 21 (with a magnified designated area) after cell seeding. Green, red, and blue fluorescence channels represent live cells, dead cells, and nuclei, respectively. Color enhancement was applied using Zen lite software (maximum intensity) for better visualization. Scale bars = 50 µm; (**B**) quantitative assessment of the cell viability at days 1, 7, and 21; (**C**) light microscopy images of Alizarin Red S staining of scaffolds for visualization of mineralization progress at day 21; (**D**) ALP activity (in U/mL) of the rat MSCs on days 1, 7, 14, and 21. Statistically significant differences are indicated as *, **, and *** according to the *p* < 0.05.

To assess the impact of HAp on cell viability, rat MSC-seeded scaffolds were cultured for 3 weeks. Figure 4A shows the combined confocal laser-scanning microscope images of the 0%, 5%, and 10% HAp-containing scaffolds on days 1, 7, and 21 after cell seeding, in which green, blue, and red fluorescence channels correspond to live cells, nuclei, and dead cells, respectively. The dominant green fluorescence in all scaffolds indicates excellent colonization and high cytocompatibility for all three scaffold types. The morphology of the cells is a critical factor in representing their attachment as well as their interactions with the matrix and other cells, all of which are crucial elements in assessing the functionality of the cells [43].

On day 1, the cells within the scaffolds appear predominantly spherical, with minimal visible cytoplasm in the imagery and a sparse presence of more elongated cells. Over time, cell density increases, indicating that the cells retain their functional properties, including the ability to divide and sustain normal metabolic activity. After 7 days, cells on all scaffolds demonstrate significant proliferation and formation of homogeneous matrices, with elongated cell morphology, indicating the proper attachment of the cells to the supporting matrix, a comfortable environment, and proceeding differentiation. On day 21, whereas 0% and 5% HAp scaffold samples exhibit an expanded network of interconnected cells, the 10% HAp sample exhibits more cell-free regions and a higher number of round-shaped cells. These observations may indicate a cellular response of osteoblasts to stress in the 10% HAp samples.

It has been reported that the excessive rigidity of HAp-containing scaffolds at later stages of bone tissue formation or a detained degradation rate may lead to stress and detachment of differentiated MSCs from the matrix [44,45,46]. However, the reported critical values of the elastic moduli that can lead to stress have exceeded 150 kPa. Therefore, excessive rigidity is unlikely to be the reason for stress in the case of 10% HAp. The quantitative assessment of the cytocompatibility showed that all cryogels sustained high cell viability, demonstrating an increase from 92 to 95% on day 1 to 95–96% by day 21, Figure 4B, with no significant difference among the samples. The obtained viability indicated excellent and comparable environmental conditions provided by the Gel/OxAlg-based scaffolds for maintaining metabolic activity. The statistical analysis of cell viability did not reveal any correlation with HAp content or physical/mechanical characteristics of the scaffolds.

Alizarin Red S staining, performed on day 21, Figure 4C, revealed successful mineralization and calcium deposition nodules in all samples, yet with a notably higher color intensity in the 5% and 10% HAp scaffolds, indicating a higher degree of mineralization compared with the HAp-free sample. We associated the induced mineralization of HAp-containing scaffolds with an increased rigidity, which positively affects cell adhesion, proliferation, and differentiation. Despite the round-shaped cellular morphology found on 10% HAp scaffolds after three weeks, which may indicate suboptimal conditions or stress, the obtained results align with previous studies that reported the acceleration of mineralization and bone tissue formation in HAp-doped hydrogel scaffolds [47,48,49].

Alkaline phosphatase (ALP) is a critical enzyme involved in the breakdown of extracellular inorganic pyrophosphate (PPi) into inorganic phosphate (Pi), facilitating the deposition of calcium phosphate minerals in the extracellular matrix, which is essential for bone mineralization and formation [50]. The successful osteogenic differentiation of rat MSCs on the scaffolds was verified by measuring the ALP activity on days 1, 7, 14, and 21, Figure 4D. A comparison between the initial enzymatic activity and its levels over 3 weeks provided interim insights into the cell differentiation process and helped to verify ongoing cellular activity. The differences in the initial ALP activity among scaffolds can provide early insights into the biological activity of the scaffolds and interactions of cells with the matrix.

The initial ALP activity was significantly higher in the 5% HAp samples compared to the HAp-free and 10% HAp constructs. This can be attributed to the enhanced osteogenic activity and higher stiffness of the HAp-doped scaffolds, which promote better cell adhesion and consequently result in higher ALP levels [51,52,53]. The stiffness alone and the osteogenic bioactivity of HAp cannot fully explain the lower ALP values observed in the 10% HAp samples compared to the 5% HAp samples during the first week. This discrepancy suggests that the bioactivity is also influenced by the morphological differences in these scaffolds. Specifically, the 10% HAp samples exhibited a smoother surface with reduced thickness, while their pores were more elongated and stretched. These morphological variations likely contributed to the differences in ALP activity observed between the two concentrations [54,55,56].

After 7 days, all scaffolds demonstrated a moderate increase in ALP activity, though the increase was less pronounced for the 10% HAp. This elevation in ALP suggests successful MSC differentiation into osteoblasts, secretion of ECM, and the initiation of mineralization across all scaffolds. By two weeks, ALP activity began to gradually decrease, continuing until day 21, while cell viability slowly increased. These results may indicate faster osteoblast maturation, leading to reduced enzymatic activity. This trend aligns with the reports in the literature of peak ALP activity around days 5–7, followed by decreased activity as differentiation progresses and mineralization occurs [57,58].

## 3. Conclusions

An innovative approach in which 3D-printed constructs undergo cryogenic crosslinking enables the fabrication of highly biocompatible hydrogel scaffolds with exceptionally large pore sizes exceeding 200 µm, which are beneficial for bone tissue regeneration applications. In this study, we assessed the effect of HAp on the mechanical and biological characteristics of magnoporous 3D-printed scaffolds produced from 2.86% Gel/OxAlg-based ink. Scaffolds with 5% and 10% of HAp were fabricated via 3D printing and evaluated for morphology, shape fidelity, degradation rate, mechanical strength, cytotoxicity, and effectiveness in mineralization and compared to the HAp-free hydrogel constructs.

Our findings show that 2.86% Gel/OxAlg ink doped with 5% HAp demonstrated increased viscosity and a notable contribution to elastic behavior, improving printability and post-printing shape fidelity. In contrast, a 10% HAp concentration led to a significant reduction in viscosity, particularly at lower shear rates, resulting in a compromised shape fidelity compared to both 0% HAp (control) and 5% HAp scaffolds. Mechanical and morphological characterization of the fabricated scaffolds revealed that 5% and 10% HAp-doped scaffolds demonstrated a notable decrease in average pore size compared to HAp-free constructs while showing enhanced stability and comparable delayed degradation rates. Additionally, we showed that the rigidity of the scaffolds improved with higher HAp content, demonstrating an increase in compression modulus from 11 kPa for the HAp-free scaffold to 18 kPa and 21 kPa for the 5% and 10% HAp-doped scaffolds, respectively.

The biological activity and cytocompatibility of the scaffolds were assessed using rat MSCs seeded on the scaffolds and cultivated for 3 weeks. All scaffolds showed excellent cytocompatibility, as evidenced by high cell viability, successful proliferation, differentiation of mesenchymal cells into osteoblasts, ECM formation, and calcification, as monitored via fluorescent live/dead assay, APL activity, and Alizarin Red S staining. However, 5% and 10% of HAp-containing structures demonstrated an enhanced degree of calcification compared to the HAp-free control scaffolds. These results indicate that adding 5% HAp to the 2.86% Gel/OxAlg improved the scaffolds’ fabrication accuracy, stability, and mechanical strength. We showed that optimization of the dopant concentration can improve the mechanical and biological qualities of scaffolds, maximizing their potential in bone tissue regeneration applications.

To further improve scaffold materials for specific applications, we continue to explore the effects of additional organic and inorganic dopants and polymeric materials on fabrication accuracy, mechanical properties, and biological performance.

## 4. Materials and Methods

### 4.1. Materials

ALP kit (ab83369), Hoechst 33,342 (ab228551), ethidium homodimer (ab145323), and Calcein AM (ab141420) were obtained from Abcam (Cambridge, UK).

β-glycerophosphate disodium salt hydrate, 99% (G9422), sodium periodate (311448), HAp (nanopowder < 200 nm particle size (BET) ≥ 97%, synthetic), NaCl (S9888), CaCl_2_, 96% (793639), polyfreeze (P0091), PBS (P4417), dexamethasone (D2915), sodium bicarbonate (S6014), ethylene glycol (324558), alginic acid sodium salt (A2033), L-ascorbic acid 2-phosphate sesquimagnesium salt hydrate, 95% (A8960), DPX mountant (06522), trypan blue (T8154), DMSO (472301), high-glucose DMEM (D6429), MTT (11465007001), non-essential amino acid solution 100X (#M7145), Alizarin Red S (130-22-3), sucrose (S7903), and gelatin from porcine skin (G2500) were obtained from Sigma-Aldrich (Saint Louis, MO, USA).

Eosin-Y (10047101), antibiotic-antimycotic (HYCLSV30079.01), and hematoxylin (3870.1000) were obtained from VWR Chemicals. The 0.5% trypsin-EDTA solution 10X (sc-363354) was obtained from ChemCruz. Fetal bovine serum (FBS-HI-22A) was obtained from Capricorn Scientific. Glutaraldehyde, 50% in H_2_O (4995.1) was obtained from Carl Roth, glycine (1610718) was obtained from Bio-Rad, and sodium borohydride (S0480) was obtained from Tokyo Chemical Industry (Tokyo, Japan). All chemicals were utilized as received, without additional purification.

Rat mesenchymal stem cells (rMSCs) sourced from the bone marrow of healthy animal donors were supplied by the National Center of Biotechnology, Kazakhstan (Astana, Kazakhstan). The isolation procedure adhered to the guidelines of the Declaration of Helsinki and received approval from the Local Ethics Committee and the Institutional Review Board of the National Center for Biotechnology (IRB 00013497).

### 4.2. Synthesis of Oxidized Alginate (OxAlg)

Oxidized sodium alginate (OxAlg) was produced by oxidizing vicinal diols through periodate cleavage, resulting in the formation of two aldehyde groups [17]. For achieving a 10% oxidation level, 0.0227 mol of sodium alginate was dissolved in ultrapure water (165 mL) and subjected to a reaction with 2.27 mmol of sodium periodate under stirring in a dark environment overnight. The reaction was subsequently halted by introducing 1 mL of ethylene glycol and the product was precipitated by adding 20 mL of 1M NaCl to the reaction flask. The obtained OxAlg was purified by re-precipitation from a water–ethanol solution three times. The product was lyophilized and stored at 4 °C in the dark. An 8% *w*/*v* OxAlg aqueous solution was utilized as a stock for subsequent ink preparation. The successful synthesis of OxAlg was verified by FTIR analysis (Nicolet iS10; Thermo Fisher Scientific, Waltham, MA, USA) (Figure S1).

### 4.3. Ink Formulations

The ink was formulated based on a previously described composition [18]. Briefly, 1 mL of a 1:1 mixture of 8% *w*/*v* aqueous solutions of gelatin (Gel) and OxAlg was diluted with 1.8 mL ultrapure water containing 4 µM glutaraldehyde. The 2.86% Gel/OxAlg ink was doped with 5% or 10% w/dry polymer mass HAp nanoparticles and homogenized. An undoped formulation was used as a reference in this study. All components were sterilized under UV light for 24 h before use. Table 1 indicates the composition of the inks.

### 4.4. Rheology Tests

The rheological behavior of the hydrogel inks was assessed using an MCR302 rheometer (Anton Paar, Austria). For these tests, a parallel plate with a diameter of 20 mm was employed. A rotational test at 15 °C was performed to evaluate viscosity across shear rate range (0.01 to 100 s^−1^). The profiles of the storage (G′) and loss (G′) moduli across a range of temperatures (10–40 °C) were transformed into a damping factor, tan (δ), defined as the ratio of G″ to G′. The measurement was conducted at a steady shear strain (γ) of 1% and a constant frequency of 1 s^−1^, with a heating rate set at 1.01 °C per min.

### 4.5. Optimization of Printing Conditions

Comprehensive optimization of several key printing parameters, such as fiber thickness, printing speed, and temperature, was performed to achieve maximum printing accuracy. This parameter played a critical role in determining the precision and quality of printed scaffolds. To ensure the post-production integrity of magnoporous scaffolds, 0.45 mm thick fibers were selected as optimal. For this purpose, a syringe equipped with a 27 G gauge needle was used. The conditions under which the desired fibers were unimpededly extruded were optimized separately for each ink composition, Table 2.

### 4.6. Fabrication of 3D Scaffolds

All scaffolds were fabricated using a BIOX 3D printer (CELLINK, Gothenburg, Sweden) equipped with a temperature-controlled printing bed and a print head. A syringe pre-loaded with the respective ink was installed in the printer, and 15 consecutive layers were deposited on a glass substrate maintained at 15 °C to maximize post-printing shape fidelity. The printing speed, extrusion pressure, and printing temperature were adjusted according to their composition, as indicated in Table 2. After printing, the scaffolds underwent a 24 h cryogelation cycle at −20 °C. Subsequently, the scaffolds were thawed, treated with sodium borohydride to reduce the Schiff bases and unreacted aldehyde groups formed during cryogelation, and thoroughly washed in TDW. After 24 h of lyophilization, the scaffolds were stored at 4 °C until use.

### 4.7. Characterization of Scaffolds

#### 4.7.1. Morphology

After lyophilization, the investigated samples were sectioned using a razor blade, and morphological analysis of the 3D scaffolds was conducted using scanning electron microscopy (SEM) on a JSM-IT200 (Jeol, Tokyo, Japan) at an accelerating voltage of 5 kV, using 7 nm thick Au coating. Pore sizes were analyzed using SEM images and ImageJ binary grayscale surface analysis. SEM images of the four samples were analyzed for each type of scaffold based on 2 mm × 2 mm sections. The obtained values were averaged.

#### 4.7.2. Swelling Test

The initial mass of the respective lyophilized cryogel scaffolds was recorded and compared to the mass after 1 h and 6 h of submerging in 10 mM PBS (pH 7.4). The swollen scaffolds were placed on a glass slide and weighed individually after removing the excess buffer. Swelling capacity was calculated using Equation (1) [26]:

(1) $$Swelling\;capacity\;\%\left(SC\right)=\frac{W\left(t\right)-W\left(0\right)}{W\left(0\right)}\times 100\%$$

where *W*(*t*) is the weight of the scaffold after incubation in PBS and *W*(0) is the dry weight of the lyophilized scaffold.

#### 4.7.3. Degradation Test

The degradation rate of the 3D-printed scaffolds was determined at 37 °C in a 5% CO_2_ atmosphere and calculated using Equation (2), where *W*(0) is the initial weight of the freeze-dried scaffolds and *W*(*f*) is the weight at a certain time point within the 21 day test period (N = 12 samples per composition). The scaffolds were immersed in a medium of DMEM with 10% FBS and 1% penicillin–streptomycin. The medium was refreshed thrice weekly. Selected scaffolds (N = 3) were retrieved on days 1, 7, 14, and 21, removed from the medium, lyophilized, and weighed again to obtain the final weight. This process was carried out individually for three different scaffold compositions.

(2) $$Degradation\;degree\;\%\left(DD\right)=\frac{W\left(0\right)-W\left(f\right)}{W\left(0\right)}\times 100\%$$

#### 4.7.4. Shape Fidelity

The accuracy of the construct printing, shape fidelity (SF, %) was evaluated by comparing the dimensions of the printed structure with those specified in the computer-aided design (CAD) model. The printing accuracy percentage was calculated utilizing Equation (3), based on the surface area (A) of the voids (N = 48) within a single scaffold. The calculation was based on the ratio of the actual and theoretical surface areas of voids (avoid). The results were averaged for each scaffold type with three samples per type (N = 3).

(3) $$\;Shape\;fidelity\;\%\;(SF)=\frac{\sum [A_{void}^{theor}-\sqrt{(A_{void}^{pract}}{-A_{void}^{theor})}^{2}]}{\sum A_{void}^{theor}}\times 100$$

where $A_{void}^{theor}$ and $A_{void}^{pract}$ denote the surface areas of theoretical and actual voids in a single scaffold, respectively.

#### 4.7.5. Mechanical Testing

The elastic modulus of the scaffolds was measured with a TA.XTplusC texture analyzer (Stable Micro Systems, Godalming, UK), which was equipped with a 0.5 kg load cell and a 50 mm diameter plunger, controlled by Exponent Connect software (version 8.0). The plunger compressed each scaffold at a rate of 1 mm/s until 45% deformation was reached. Three samples of each scaffold type were tested, and the resulting values were averaged and displayed graphically. The elastic modulus (E) was determined from the linear portion (0–10%) of the stress–strain curve using Equation (4):

(4) $$E=\frac{\frac{F}{A}}{\frac{∆h}{h}}$$

where *h* represents the initial height; ∆*h* represents the change in height under compression; *F* denotes the applied force; and *A* is the cross-sectional area of the scaffold. Each test was conducted on three samples (N = 3) for accuracy, and the obtained results were averaged.

### 4.8. Cell Culture

Rat mesenchymal stem cells (rMSCs) were cultured according to the protocol established by Zhang et al. [59]. The expansion of rMSCs was carried out over seven days in a growth medium comprising DMEM, 10% FBS, 1% NEAA, 1% penicillin/streptomycin, and 0.001% of bFGF. The expansion medium was renewed every two–three days. A 0.25% trypsin EDTA solution was used for cell detachment, with an incubation time of 3 min. The osteogenic medium that was used for the induction of cell differentiation, 500 mL of complete medium, contained 500 μL of dexamethasone (100 nM), 10 mL of β-glycerophosphate disodium salt hydrate (10 mM), and 500 μL of L-ascorbic acid-2-phosphate sesquimagnesium salt hydrate (50 μg/mL). The cells were kept in a humidified atmosphere at 37 °C and 5% CO_2_.

### 4.9. Cell Seeding and Cell Viability

Following a week of cultivation, cells were detached from the culture flask using 0.25% trypsin-EDTA and counted with a hemocytometer. They were then seeded onto the scaffolds at a density of 5 × 10^5^ cells per scaffold. Prior to this, the scaffolds were sterilized with ethanol, rinsed with PBS, and soaked in complete medium. The scaffolds were then incubated for 2 h to allow for cell adhesion.

For the assessment of cell morphology and viability, the scaffolds were imaged on days 1, 7, and 21 using a confocal laser-scanning microscope (LSM 780; ZEISS, Oberkochen, Germany). Preceding imaging, the scaffolds were washed with PBS and stained in complete medium using fluorescent dyes: Hoechst 33,342 for nuclei, Calcein AM for live cells, and ethidium homodimer for dead cells. After staining, the scaffolds were incubated for 40 min at 37 °C, washed with PBS, and placed in imaging chambers (ibidi, μ-Slide) with complete medium. Images were captured with a ×10 objective lens using the excitation channels: 350/461 nm (Hoechst), 404/517 nm (calcein), and 517/617 nm (ethidium homodimer). Live and dead cells were then counted using ImageJ (Fiji) software, and cell viability was calculated using Equation (5).

(5) $$Cell\;viability\;(\%)=\frac{\#Live\;}{\#Live+\#Dead}\times 100\%$$

Enhanced color settings were applied to improve visualization (using maximum intensity).

### 4.10. Alkaline Phosphatase Activity

Osteogenic media extracted from the scaffolds were gathered on days 1 and 7 to assess alkaline phosphatase (ALP) levels. An ELISA kit was used to determine the ALP concentration following the standard procedure. In brief, the collected samples were placed on a 96-well plate, diluted with an assay buffer, and 5 mM p-nitrophenyl phosphate (pNPP) was added. The interaction between ALP and pNPP resulted in the formation of yellow p-nitrophenol. Each sample was analyzed in quadruplicate. Additionally, six standards were prepared, ranging from 0 to 50 µL of 1 mM pNPP mixed with assay buffer, and added to the wells containing the ALP enzyme solution. The standards were analyzed in duplicate. The plate was then covered with foil and incubated at room temperature for one hour. After incubation, a stop solution was added to all wells, and the absorbance was measured at an optical density (OD) of 405 nm using a microplate reader.

### 4.11. Histology

After a 21-day culture period, the cell-laden scaffolds were subjected to histological analysis and evaluated for calcium accumulation and ECM mineralization using Alizarin Red S staining.

The samples were prepared by rinsing the scaffolds with a solution of 10 mM CaCl_2_ and 0.15 M NaCl. The scaffolds were subsequently fixed in a 4% paraformaldehyde solution at room temperature for 2 h. After fixation, the scaffolds were washed twice with the same CaCl_2_/NaCl solution and soaked in a 10% sucrose solution with 10 mM CaCl_2_ for 2 h at room temperature. This was followed by immersion in a 30% sucrose solution with 10 mM CaCl_2_ for 16 h. The scaffolds were rapidly frozen in cryomolds using optimal cutting temperature (OCT) compound and liquid nitrogen, then stored at −80 °C for subsequent cryosectioning. Sections were prepared using a CryoStar NX70 cryotome (Thermo Fisher Scientific). The cut 15 µm thick slices were placed on adhesive polysine slides. The slides were air-dried for 10 min and then stored at −20 °C until needed.

The slices were mounted on glass slides and washed with TDW to remove OCT compounds. The samples were then stained with Alizarin Red S solution (pH 4.2) for two minutes, followed by dehydration using sequential rinsing with acetone, an acetone-xylene mixture (1:1 ratio), and xylene. The samples were sealed with DPX mounting medium and covered with glass slides (18 mm, 0.13–0.17 mm thick). Images were obtained using an inverted field microscope (Jenco, Huber Heights, OH, USA).

### 4.12. Statistical Analysis

Statistical evaluations were conducted using the Origin software 2022 (version 9.9). Quantitative results are presented as mean ± standard deviation (SD). For data comparison, ANOVA was applied, followed by Tukey’s test for individual pairwise comparisons. A *p*-value of less than 0.05 was deemed indicative of statistical significance.

## Figures and Tables

**Table 1:** Compositions of the formulated inks.

Ink Composition	Gel (mg/mL)	OxAlg (mg/mL)	HAp (mg/mL)
Gel/OxAlg/0% HAp	28.6	28.6	0
Gel/OxAlg/5% HAp	28.6	28.6	2.86
Gel/OxAlg/10% HAp	28.6	28.6	5.72

**Table 2:** Optimized printing conditions for Gel/OxAlg/HAp inks.

Ink	Extrusion Pressure (kPa)	Print Head Speed (mm·s^−1^)	Print Head Temperature (°C)	Print Bed Temperature (°C)
2.86% Gel/OxAlg/0% HAp	68–70	5	12.5	15
2.86% Gel/OxAlg/5% HAp	75–78	5	11.5	15
2.86% Gel/OxAlg/10% HAp	78–82	5.5	12.5	15

## Data Availability

All data and materials are available on request from the corresponding author. The data are not publicly available due to ongoing research using a part of the data.

## Supplementary Materials

The following supporting information can be downloaded at: https://www.mdpi.com/article/10.3390/gels10060406/s1, Figure S1: FTIR spectra of both oxidized and untreated alginate. The arrow points to the 1730 cm^−1^ region, which corresponds to the C=O bond stretching in the aldehyde group.
