# Supplementary material for: Impact of Hydroxyapatite on Gelatin/Oxidized Alginate 3D-Printed Cryogel Scaffolds
Source: Gels. 2024 Jun 18;10(6):406. doi: 10.3390/gels10060406 (PMC11203254; doi:10.3390/gels10060406)
Supplement: Supplementary file 1 [file gels-10-00406-s001.zip › gels-3059282-supplementary.pdf]

Impact of Hydroxyapatite on Gelatin/Oxidized Alginate 3D-Printed Cryogel Scaffolds

Supporting Information

Ainur Zhanbassynova[1], Fariza Mukasheva[1], Madi Abilev[1], Dmitriy Berillo[2], Alexander Trifonov[1], and Dana Akilbekova[1]*

[1]Department of Chemical and Materials Engineering, School of Engineering and Digital Sciences, Nazarbayev University, 010000 Astana, Kazakhstan.

[2]Department of Chemistry and Biochemical Engineering, Satbayev University, 050013 Almaty, Kazakhstan.

*Corresponding author, email: dana.akilbekova@nu.edu.kz

Keywords: 3D printing, cryogelation, hydroxyapatite, stem cells, bone tissue engineering.

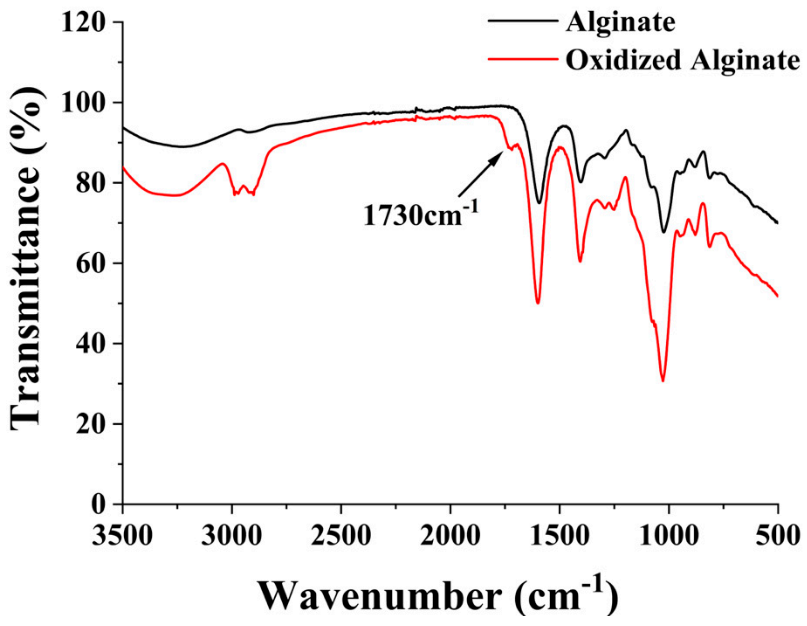

Figure S1: FTIR spectra of both oxidized and untreated alginate are shown. The arrow points to the 1730 cm^{-1} region, which corresponds to the C=O bond stretching in the aldehyde group, signifying the successful synthesis of oxidized alginate.
